# Composition of *Anopheles* mosquitoes, their blood-meal hosts, and *Plasmodium falciparum* infection rates in three islands with disparate bed net coverage in Lake Victoria, Kenya

**DOI:** 10.1186/s12936-017-2015-5

**Published:** 2017-09-08

**Authors:** Edwin Ogola, Jandouwe Villinger, Danspaid Mabuka, David Omondi, Benedict Orindi, James Mutunga, Vincent Owino, Daniel K Masiga

**Affiliations:** 1 International Centre of Insect Physiology and Ecology (icipe), P.O. Box 30772, Nairobi, 00100 Kenya; 2 Department of Biochemistry and Molecular Biology, Egerton University Njoro Campus, P.O. Box 536, Egerton, 20115 Kenya

**Keywords:** Malaria vector, Blood-meal, Malaria parasite, *Plasmodium falciparum*, Malaria transmission

## Abstract

**Background:**

Small islands serve as potential malaria reservoirs through which new infections might come to the mainland and may be important targets in malaria elimination efforts. This study investigated malaria vector species diversity, blood-meal hosts, *Plasmodium* infection rates, and long-lasting insecticidal net (LLIN) coverage on Mageta, Magare and Ngodhe Islands of Lake Victoria in western Kenya, a region where extensive vector control is implemented on the mainland.

**Results:**

From trapping for six consecutive nights per month (November 2012 to March 2015) using CDC light traps, pyrethrum spray catches and backpack aspiration, 1868 *Anopheles* mosquitoes were collected. Based on their cytochrome oxidase I (*COI*) and intergenic spacer region PCR and sequencing, *Anopheles gambiae* s.l. (68.52%), *Anopheles coustani* (19.81%) and *Anopheles funestus* s.l. (11.67%) mosquitoes were differentiated. The mean abundance of *Anopheles* mosquitoes per building per trap was significantly higher (p < 0.001) in Mageta than in Magare and Ngodhe. Mageta was also the most populated island (n = 6487) with low LLIN coverage of 62.35% compared to Ngodhe (n = 484; 88.31%) and Magare (n = 250; 98.59%). Overall, 416 (22.27%) engorged *Anopheles* mosquitoes were analysed, of which 41 tested positive for *Plasmodium falciparum* infection by high-resolution melting (HRM) analysis of 18S rRNA and cytochrome b PCR products. *Plasmodium falciparum* infection rates were 10.00, 11.76, 0, and 18.75% among blood-fed *An. gambiae* s.s. (n = 320), *Anopheles arabiensis* (n = 51), *An. funestus* s.s. (n = 29), and *An. coustani* (n = 16), respectively. Based on HRM analysis of vertebrate cytochrome b, *16S* rRNA and *COI* PCR products, humans (72.36%) were the prominent blood-meal hosts of malaria vectors, but 20.91% of blood-meals were from non-human vertebrate hosts.

**Conclusions:**

These findings demonstrate high *Plasmodium* infection rates among the primary malaria vectors *An. gambiae* s.s. and *An. arabiensis*, as well as in *An. coustani* for the first time in the region, and that non-human blood-meal sources play an important role in their ecology. Further, the higher *Anopheles* mosquito abundances on the only low LLIN coverage island of Mageta suggests that high LLIN coverage has been effective in reducing malaria vector populations on Magare and Ngodhe Islands.

## Background

Small island communities are harder to reach than mainland populations and do not have the same access to malaria control interventions and are less researched. Malaria continues to be a major public health problem and a key impediment to socio-economic development in areas around Lake Victoria, western Kenya where recent studies have focused on the mainland [1–4] and large islands, such as Rusinga Island [5, 6]. Despite higher control efforts in these regions, traffic from these small islands might sustain transmission if not equally targeted. The Lake Victoria island region provides suitable habitats for abundant and diverse anophelines that support efficient endemic malaria transmission all year round [7, 8], with various health facilities reporting more than 40% prevalence rates based on rapid diagnostic tests (RDTs) at public hospitals [9]. The main anophelines present in this region are *Anopheles gambiae* sensu stricto (s.s.) and *Anopheles arabiensis*, with *Anopheles funestus* bridging transmission during dry seasons and *Anopheles coustani* as a secondary vector [7, 10, 11].

The current front-line strategies for malaria control in this region are malaria patient management [12], following the WHO strategy of diagnostic testing, treatment and malaria surveillance [13], and protecting people from receiving infectious bites through long-lasting insecticidal nets (LLINs) [14]. LLINs have been used extensively to reduce mosquito density and biting activity, causing a decline in malaria transmission in the Lake Victoria region of western Kenya [14–16]. Despite the decline, malaria transmission remains, undermining the current drive to eliminate malaria [17].

Local transmission can be maintained by importation of infection through movement of infected people and/or mosquitoes within an endemic area [18, 19], creating malaria hotspots that facilitate widespread transmission [20]. Therefore, monitoring localized transmission dynamics is important in determining actual effectiveness of control strategies deployed, and can guide appropriate adjustments. Malaria control interventions can be more effective if applied based on knowledge of geographically localized transmission dynamics as influenced by vector species and their blood-meal sources. This study was undertaken to investigate LLIN coverage and compare the distribution of malaria vectors, blood-meal sources, and *Plasmodium* infection rates in three small islands with neglected communities in Lake Victoria, western Kenya.

## Methods

### Study location

**Fig. 1:**
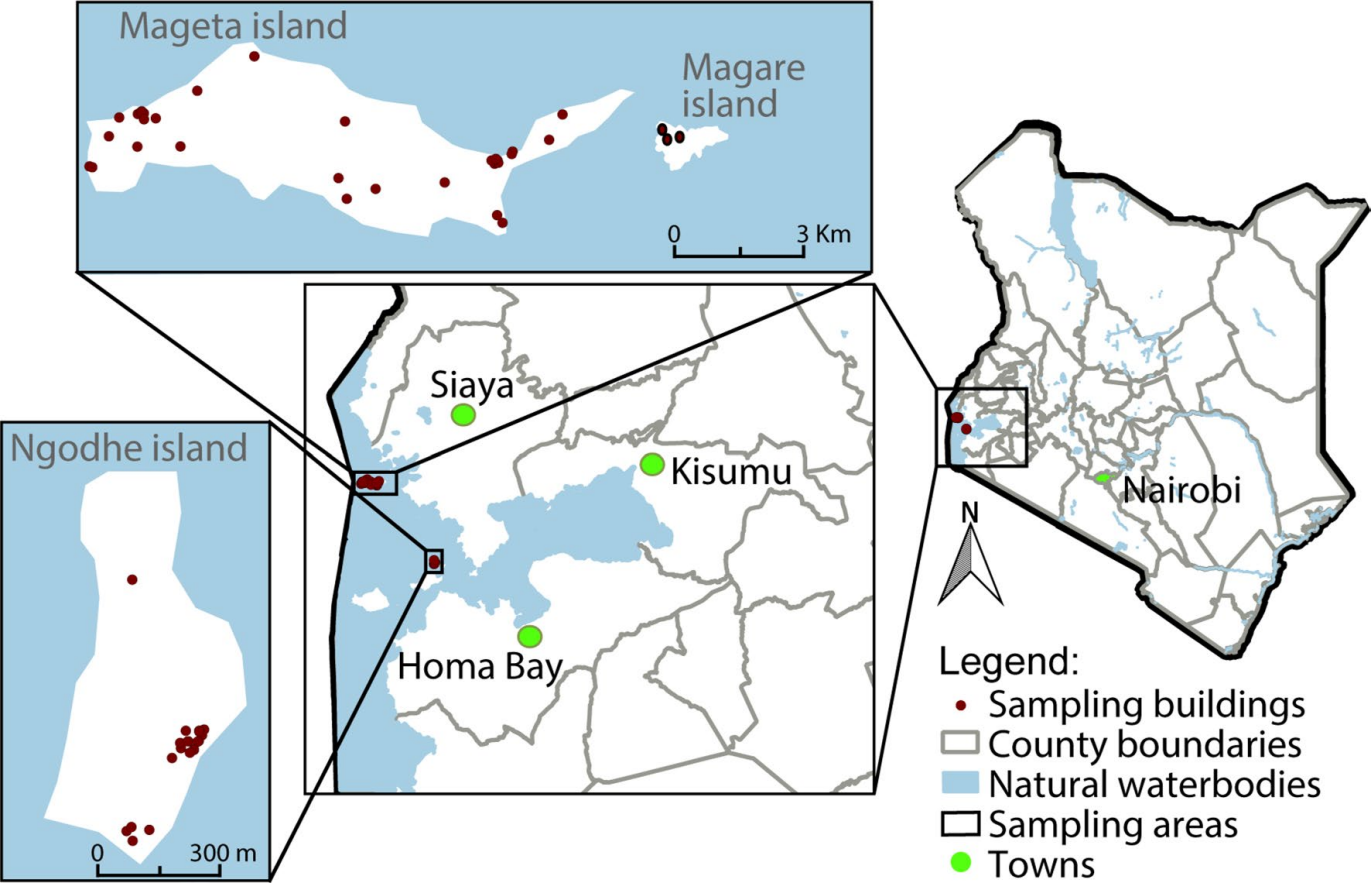
Map showing study locations. The three study islands are located in Lake Victoria, western Kenya. Mageta and Magare are located in Siaya County, while Ngodhe is in Homa Bay County. Areas of Mageta, Magare and Ngodhe are 7.02, 0.20 and 0.90 sq km, respectively. The distance between Mageta and Magare Islands is about 0.30, which are about 28.5 km north-west of Ngodhe Island

The study was conducted in Mageta, Magare and Ngodhe Islands of Lake Victoria in western Kenya (Fig. 1). Mageta and Magare Islands are in Siaya County, while Ngodhe Island is in Homa Bay County. The sizes of Mageta, Magare and Ngodhe are approximately 7.02, 0.20 and 0.90 sq km, respectively, while the distance between Mageta and Magare is about 0.30 km, and Ngodhe is about 28.50 km from Mageta. Mageta and Magare are about 7.5 km from the mainland, while Ngodhe is about 3 km from a relatively larger island (Rusinga). The islands are only accessible by ferry services or boats. In addition, all three islands have a rocky terrain. Therefore, the islands are difficult to reach when compared with the mainland. The main economic activities in the islands are fishing, fishmongering and small-scale agriculture.

### Household information

All buildings from which mosquitoes were samples on the three islands, including unoccupied mills that were part of residential complexes, were identified and geo-referenced using a hand-held geographical positioning system (eTrex, Vista, Garmin, USA). The roofing and wall types were recorded and the nature of eaves scored as either opened or closed. With the household head/adult member (i.e., aged over 18 years) of the household as the respondent, a survey was carried out to determine island populations and LLIN coverage (the percentage of households having at least one LLIN).

### Adult mosquito trapping

Adult mosquitoes were trapped indoors or outdoors from 16 geo-referenced residential buildings on each island for six consecutive nights each month between November 2012 to September 2014 (23 months; 2208 trap nights) in Ngodhe, and June 2013 to March 2015 (22 months; 2112 trap nights) in Magare and Mageta. Residential buildings were defined as housing units comprising one or two people sharing a living space. When members of a family lived in the same compound with several buildings, each building was regarded as a separate residential building if at least one member of the family spends the night in the housing unit. One randomized trapping method (unbaited indoor or outdoor CDC light traps, indoor pyrethrum spray catches (PSC) or indoor backpack aspiration (ASP) was used per geo-referenced residential building in a sampling month. In Magare and Ngodhe, buildings were resampled across months (with different methods) due to the limited availability of residential houses on these islands. The trapping nights were selected to coincide with the period before full moon [21, 22]. Overall, 96 trappings were done per month on each island, with Ngodhe having an additional trapping month of 96 collections. Host-seeking vectors were trapped using unbaited CDC light traps set indoors or outdoors in the evening at 18:00 and removed the following morning at 06:00. The CDC indoors light traps were set on the foot side of a person’s bed (about 1.5 m from the ground), irrespective of whether they were sleeping under a bed net or not. Outdoors, CDC light traps were set about 20 m from a sampling building and a present cattle shed. Indoor-resting vectors were collected using PSC and ASP between 06:00 and 08:00.

After collection, mosquitoes were anaesthetized with chloroform [23], identified using morphological keys and sexed [24]. All the females belonging to *An. gambiae, An. funestus* and *An. coustani* species complexes were counted and classified on the basis of their abdominal status as blood-fed (engorged), gravid, half-gravid, or unfed (not engorged) [25]. Further, the females were preserved individually in barcoded vials containing isopropanol, stored at room temperature in the field and at −20 °C in the laboratory for further analysis. The labelling indicated details of collection method, building identification, site identification, morphological identification, sex and collection date.

### Nucleic acid extraction

The engorged abdomens of field-collected and laboratory-reared adult anophelines were separated from the rest of the body (head, thorax, legs) using sterile forceps and dissection pins, and transferred into individual sterile 1.5-mL microtubes. Genomic DNA from the engorged abdomens, remaining body parts (head, thorax, legs), and known vertebrate whole blood samples were extracted separately using DNeasy Blood and Tissue Kit (QIAGEN, Hilden, Germany) following the manufacturer’s extraction protocol with some modifications. Briefly, 200 µL of PBS (phosphate-buffered saline, pH 7.4) was added to the sample in a 1.5-mL microcentrifuge tube containing 20 µL proteinase K and the sample mixed by vortexing for 15 s. Then, 200 µL lysis buffer was added, vortexed and the mixture incubated at 56 °C for 2 h. Subsequently, 200 µL of absolute ethanol was added. The solution was mixed by vortexing and homogenate transferred into a mini spin column placed in a 2-mL collection tube before centrifugation for 1 min at 6000×*g*. The flow-through and collection tubes were discarded and the spin column transferred to a new 2-mL collection tube, 500 µL of buffer AW1 was added and centrifuged for 3 min at 20,000×*g*. The flow-through and the collection tubes were discarded and the spin column transferred to a new 1.5-mL micro-centrifuge tube. The DNA was finally eluted by adding 30 µL buffer AE to the centre of the spin column membrane, incubated for 3 min at room temperature (25 °C) and centrifuged for 1 min at 6000×*g*, and stored at −20 °C.

### Molecular identification of anopheline mosquitoes

Molecular identification of engorged *An. coustani*, *An. funestus* sensu lato (s.l.) and *An. gambiae* mosquitoes involved polymerase chain reaction (PCR) amplification and sequencing of the cytochrome oxidase subunit 1 (*COI*) region [26], polymorphic *ITS2* region of ribosomal DNA [27, 28] and analysing melt curve differences of 165 base pairs (bp) intergenic spacer region (*IGS*) gene amplicons obtained using *IGS* gene primers [29, 30]. Colony-reared, sugar-fed *An. gambiae* s.s. Mbita strain (established in 2001) and *An. arabiensis* Mwea strain (established in 2004) from the International Centre of Insect Physiology and Ecology (*icipe*) in Nairobi, Kenya, served as standard reference positive controls for *An. gambiae* s.l. sibling species identification and negative controls for blood-meal analysis.

Ten microlitre PCR reactions were prepared with 0.5 μM final concentrations for each primer, 2 μL of 5× Hot Firepol Evagreen HRM Mix (Solis BioDyne, Tartu, Estonia) and 1 μL of DNA template. Thermal cycling conditions for *COI* and *ITS2* were as follows: initial denaturation at 95 °C for 15 min, followed by 40 cycles of denaturation at 95 °C for 30 s, annealing at 50 °C for 30 s and extension at 72 °C for 1 min 30 s, and a final extension at 72 °C for 7 min. For *IGS* amplification, the conditions were as follows: initial denaturation at 95 °C for 15 min, followed by 40 cycles of denaturation at 95 °C for 30 s, annealing at 57 °C for 30 s, and extension at 72 °C for 45 s and a final extension at 72 °C for 7 min. PCR reactions for *COI* and *ITS2* were conducted on Veriti thermocycler (Applied Biosystems), and for *IGS*, a high-resolution melting (HRM) capable Rotor-Gene Q real time PCR thermocycler (QIAGEN, Hilden, Germany) was used. Following PCR, HRM analysis of amplicons was conducted by gradually increasing the temperature by 0.1 °C after every 2 s from 75 to 92 °C, resulting in a plot of the change in fluorescence with time (dF/dT). PCR-HRM protocols were validated for accuracy and sensitivity using standard reference controls. ExoSAP-IT (USB Corporation, Cleveland, OH, USA) was used to remove unincorporated dNTPs and PCR primers before sequencing. Sequences were edited in Geneious 7.0.5 (http://www.geneious.com) [31] and used to query GenBank [32].

### Blood-meal source detection

High-resolution melting profiles obtained from PCR products of vertebrate cytochrome b (*cyt b*) [33–36], *16S* ribosomal (r)RNA [33] and *COI* gene primers were used to distinguish different vertebrate hosts in anopheline mosquito blood-meals. Using DNA extracted from known vertebrate whole blood as positive controls and sugar-fed colony-reared mosquito DNA extracts as negative controls, PCRs were carried out in final volumes of 10 μL, containing 6 µL of PCR water, 0.5 μM concentrations of each primer, 2 µL of 5× Hot Firepol Evagreen HRM Mix (Solis BioDyne, Tartu, Estonia) and 1 µL of DNA template. Thermal cycling conditions for *cyt b* and *16S* rRNA primers [33] were used for all engorged anophelines. The thermal cycling conditions used for *COI* primers were as follows: initial denaturation for 15 min at 95 °C, followed by 35 cycles of denaturation at 95 °C for 30 s, annealing at 50 °C for 30 s and extension at 72 °C for 60 s followed by a final extension at 72 °C for 7 min. Following PCR, HRM profile analysis of amplicons was conducted as previously stated with normalization regions between 75.0–78.0 and 88.00–95.0 °C. Known vertebrate whole blood from cow (*Bos tarsus*), pig (*Sus scrofa*), goat (*Capra hircus*), chicken (*Gallus gallus*), dog (*Canis familiaris*), and human (*Homo sapiens*) DNA collected in a previous study [33], as well as Swiss mouse (*Mus musculus*) and rabbit (*Oryctolagus cuniculus*) whole blood samples sourced from *icipe*’s animal rearing unit, served as standard reference positive controls for blood-meal analysis. Whole blood from livestock samples was obtained from a local abattoir. Blood-meal sources were identified by comparison of HRM melt curves to those of the reference control species. Amplicons with unique *cyt b*, *16S* rRNA or *COI* HRM melt curves were purified for sequencing as previously stated.

### Detection of malaria parasites

Malaria parasites in salivary glands of engorged mosquitoes were detected by analysing species-specific HRM profiles generated from PCR products of 18S rRNA [37] and 183-bp *cyt b* gene [38] primers. PCRs were carried out in final volumes of 10 μL, containing 6 µL of PCR water, 0.5 μM concentrations of each primer, 2 µL of 5× Hot Firepol Evagreen HRM Mix (Solis BioDyne, Tartu, Estonia) and 1 µL of DNA template. nPCR-HRM was used for 18S rRNA, the touchdown thermal cycling conditions [37] were used on all field-collected engorged anopheline mosquitoes. The thermal cycling conditions used for *cyt b* primers were as follows: initial denaturation for 15 min at 95 °C followed by 40 cycles of denaturation at 95 °C for 20 s, annealing at 45 °C for 60 secs and extension at 72 °C for 45 s followed by a final extension at 72 °C for 10 min. Following PCR, HRM profile analysis of amplicons was conducted as previously stated with normalization regions between 64.0–66.0 and 86.00–92.0 °C. *Plasmodium falciparum* infection was detected by comparison of melt curves to those of a standard reference positive control, *P. falciparum* DNA acquired from the National Institute for Biological Standards and Control (NIBSC; Hertfordshire, UK). Representative positive samples were purified for sequencing as previously stated. The sequences were edited in Geneious 7.0.5 software [31] and queried in GenBank using BLAST [32].

### Statistical analysis

Field entomological data were analysed using R version 3.3.0 [39]. Anopheline mosquito abundance was estimated as the mean number of *Anopheles* mosquitoes collected per building per trap. Chi square tests were used to compare these mean abundances of anophelines among the three study islands. Species composition was estimated in terms of relative abundances, the mean percentages of specific anopheline mosquito species collected per trap per building. Differences in *P. falciparum* infection rates among engorged vector species were compared in a Bayesian fashion using the Bayesian First Aid package [40]. The Bayesian approach was adopted because these data were sparse, thus rendering the classical Chi square approach for comparing proportions unreliable. Differences were considered significant if the 95% credibility interval (Bayesian equivalent of classical 95% confidence interval) did not include zero.

## Results

### Demographic information and LLIN coverage

A total of 2671 residential buildings were geo-referenced within the three islands with Mageta, Magare, and Ngodhe having 2446, 71, and 154 residential buildings, respectively (Table 1). The biggest island, Mageta, had 6487 inhabitants, while Magare and Ngodhe had 250 and 484 inhabitants, respectively. Mageta had LLIN coverage of 62% (n = 1525), which is below the universal WHO-recommended target of 80%, while Magare had 99% (n = 70) and Ngodhe had 88% (n = 136) LLIN coverage (Table 1). Most houses were made of mud walls with iron-sheet roofing and open eaves [Mageta 95% (n = 2331); Magare 100% (n = 71), Ngodhe 96.75% (n = 149)] between the wall and the roof (Table 1).

**Table 1:** Demographic information of Mageta, Magare and Ngodhe, including long-lasting insecticidal net coverage

Study island	Residential buildings	Open eaves	Population	LLIN coverage
Mageta	2446	2331	6487	1525 (62.35%)
Magare	71	71	250	70 (98.59%)
Ngodhe	154	149	484	136 (88.31%)

LLINs: long-lasting insecticide nets

### Anopheline population dynamics

**Fig. 2:**
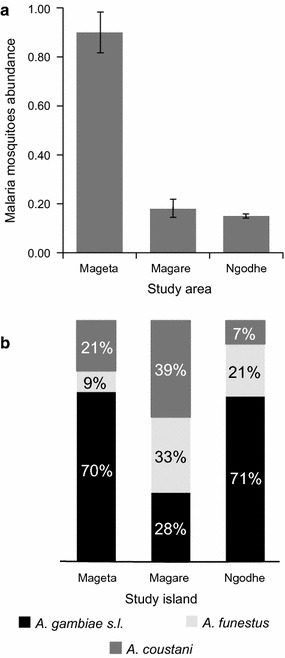
Anopheline abundance and species composition. **a** Mean abundances per trap per building of anophelines in each of the three study islands. **b** Relative abundances of malaria vector species by islands

During the study period, between November 2012 and March 2015, a total of 7350 mosquitoes were collected in pools from CDC light trap, PSC and ASP collections. Of the mosquitoes collected 2752 (37.44%) were *Aedes aegypti*, 2266 (30.83%) were *Mansonia* species and 2332 (31.73%) were anopheline species (*An. gambiae* s.l., *An. funestus* and *An. coustani*). A confirmatory *An. coustani COI* sequence was deposited into GenBank (accession MF782553). The anopheline mosquitoes collected were comprised of 464 (19.90%) males and 1868 (80.10%) females. Of the female anopheline mosquitoes sampled, 1452 (77.73%) were collected indoors and 416 (22.27%) were collected outdoors (Table 2). Of these vectors collected, 1280 (68.52%) were *An. gambiae* s.l., 370 (19.81%) were *An. coustani,* and 218 (11.67%) were *An. funestus* s.l. The mean abundance of female anophelines was significantly higher in the largest island, Mageta, compared to Magare and Ngodhe (χ^2^ = 193.26, df = 2, p < 0.001) and similar in Magare and Ngodhe (χ^2^ = 0.56, df = 1, p < 0.453) (Fig. 2a). In Mageta and Ngodhe, the predominant species in terms of relative abundance was *An. gambiae* s.l., while in Magare, which had the highest LLIN coverage, *An. coustani* was predominant (Table 2; Fig. 2b).

**Table 2:** Distribution of anophelines

Study area	Species	n (%)	Indoor												Outdoor			
			ASP				PSC				CDC				CDC			
			BF	UF	G	HG	BF	UF	G	HG	BF	UF	G	HG	BF	UF	G	HG
Mageta	*An. gambiae* s.l.	1067 (70.38)	63	35	14	41	384	53	19	81	29	267	31	7	5	35	2	1
	*An. funestus*	134 (8.84)	11	1	5	11	27	0	1	8	8	42	7	2	0	10	1	0
	*An. coustani*	315 (20.78)	0	1	0	0	0	0	0	0	4	5	0	0	35	266	4	0
	Total (%)	1516	182 (12.01)				573 (37.80)				402 (26.52)				359 (23.68)			
Magare	*An. gambiae* s.l.	20 (28.57)	0	0	1	0	4	2	0	1	0	11	0	0	0	1	0	0
	*An. funestus*	22 (31.43)	0	0	0	0	8	3	1	0	1	8	0	0	0	0	1	0
	*An. coustani*	28 (40)	0	0	0	0	0	0	0	0	0	0	0	0	2	26	0	0
	Total (%)	70	1 (1.43)				19 (27.14)				20 (28.57)				30 (42.86)			
Ngodhe	*An. gambiae* s.l.	193 (68.44)	11	18	6	3	9	11	12	2	14	60	37	4	2	2	2	0
	*An. funestus*	62 (21.99)	1	1	3	3	7	2	1	2	4	18	15	4	0	0	1	0
	*An. coustani*	27 (9.57)	0	0	0	0	0	0	0	0	1	6	0	0	2	16	2	0
	Total (%)	282	46 (16.31)				46 (16.31)				163 (57.80)				27 (9.57)			

n: number of anophelines; ASP: aspirator; PSC: pyrethrum spray collector; CDC: CDC light trap; BF: blood-fed; UF: unfed; G: gravid; HG: half gravid

### Blood-meal sources of engorged field-collected anopheline mosquitoes

The majority of 632 blood-fed (engorged) anophelines were collected indoors (92.72%, n = 586), while 7.28% (n = 46) were collected outdoors (Table 2). Of the analysed 416 (22.27%) malaria vectors, *An. gambiae* s.s. (76.92%, n = 320) was the most frequent blood-fed species, followed by *An. arabiensis* (12.26%, n = 51), *An. funestus* s.s. (6.97%, n = 29), and *An. coustani* (3.85%, n = 16) (Table 3).

**Table 3:** Number of blood-meal sources of engorged anopheline species

Study area	Species	n	Vertebrate host										
			Human	Chicken	Sheep	Cow	Goat	Pig	Frog	Rat	Dog	Bird	UN
Mageta	*An. gambiae* s.s.	310	236.5^a^	9^a^	1	25.5^a^	4^a^	2	1	5	6	1	19
	*An. arabiensis*	40	26^a^	1	4	7^a^	0	0	0	0	0	0	2
	*An. funestus* s.s.	16	15	0	0	0	0	0	0	0	0	0	1
	*An. coustani*	8	1	0	0	7	0	0	0	0	0	0	0
	Total (%)	374	278.5^a^ (74.47%)	10^a^ (2.67%)	5 (1.34%)	39.5^a^ (10.56%)	4^a^ (1.07%)	2 (0.53%)	1 (0.27%)	5 (1.34%)	6 (1.60%)	1 (0.27%)	22 (5.88%)
Magare	*An. gambiae* s.s.	7	3	0	0	1	0	0	0	1	0	0	2
	*An. arabiensis*	2	1	0	0	0	0	0	0	0	0	0	1
	*An. funestus* s.s.	8	6	0	0	0	0	0	0	0	0	0	2
	*An. coustani*	8	0	0	0	7	0	0	0	0	0	0	1
	Total (%)	25	10 (40%)	0	0	8 (32%)	0	0	0	1 (4%)	0	0	6 (24%)
Ngodhe	*An. gambiae* s.s.	3	3	0	0	0	0	0	0	0	0	0	0
	*An. arabiensis*	9	7.5^a^	0	0	1.5^a^	0	0	0	0	0	0	0
	*An. funestus* s.s.	5	2^a^	0	0	3^a^	0	0	0	0	0	0	0
	Total (%)	17	12.5^a^ (73.53%)	0	0	4.5^a^ (26.47%)	0	0	0	0	0	0	0
Total		416	297	10	5	53	4	2	2	6	6	1	27

n: numbers of engorged anophelines analysed; UN: numbers of blood-meals whose sources identification was not successful

^a^Include mixed anophelines blood-meals

**Fig. 3:**
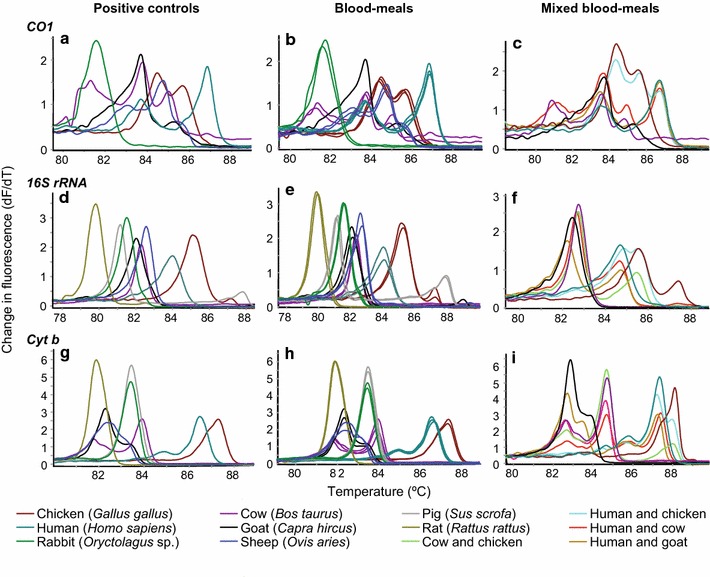
Representative melt rate profiles of anopheline blood-meal sources melt rate profiles of *COI* **a** positive controls, **b** single blood-meals, **c** mixed blood-meals, *16S rRNA* **d** positive controls, **e** single blood-meals, **f** mixed blood-meal and *cyt b* **g** positive controls, **h** single blood-meals; and, **i** mixed blood-meals

Blood-meal sources were identified from 389 *Anopheles* mosquitoes, representing 93.51% of all analysed engorged anophelines (n = 416). Overall, 10 blood-meal hosts, including humans, goats (*Capra hircus*), cows (*Bos taurus*), sheep (*Ovis aries*), dogs (*Canis lupus*), domesticated birds (chicken, *Gallus gallus*), pigs (*Sus scrofa*), rats (*Rattus rattus*), grass frogs (*Ptychadena nilotica*), and a wild bird (*Dendrocincla turdina*), were identified (Fig. 3; Table 3).

**Fig. 4:**
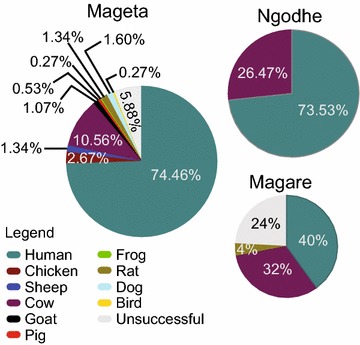
Proportion of blood-meal sources of anophelines. The pie charts show proportions of blood-meal sources among captured anophelines. The range of host species varied in all study areas

A total of 3.61% of all analysed blood-meals (n = 15) were from mixed blood-meals. In addition to humans, *An. gambiae* s.s., *An. arabiensis* and *An. funestus* had fed on cow, goat or chicken, respectively. *Anopheles gambiae* s.s. mosquitoes also fed on goat. Mixed blood-meal melt curves showed double peaks with melting temperatures similar to those of more than one positive controls (Fig. 3c, f, i). In Mageta, blood-meal sources were 74.46% human and 19.65% non-human. In Magare, 40% of blood-meal sources were from humans and 36% from other vertebrate species. In Ngodhe, 73.53% of blood-meal sources were from humans and 26.47% from non-humans. Conversely, *An. coustani*, which were predominantly collected outdoors, showed greater tendency of blood-feeding on cow (Table 3). The range of host species varied in all study areas (Fig. 4).

### Malaria parasite infection in field-collected mosquitoes

**Fig. 5:**
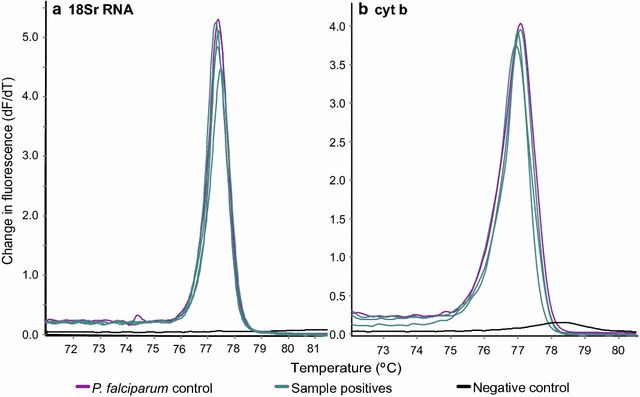
Representative melt rate profiles of *Plasmodium falciparum* control and field-collected sample positives. Melt rate profiles of **a** 18S rRNA and **b** *cyt b* PCR products. The y-axes indicate change in fluorescence units with increasing temperatures (dF/dT), with temperature shown in the x-axes. The peak melt rates represent empirical melting temperatures (Tm) *P. falciparum* PCR products

The 416 engorged anopheline mosquitoes (320 *An. gambiae* s.s., 51 *An. arabiensis,* 29 *An. funestus* s.s., 16 *An. coustani*) were further tested for presence of malaria parasites using 18S rRNA [37], and *cyt b* [38] (Fig. 5). Overall, *P. falciparum* infection rate was 9.86%. *Anopheles gambiae* s.s. had an individual *P. falciparum* infection rate of 10.00%, while the infection rate in *An. arabiensis* was 11.76%. A small number of engorged *An. coustani* analysed had a *P. falciparum* infection rate of 18.75% (Table 4). However, none of the engorged *An. funestus* samples tested positive for *P. falciparum* infection. There were no significant differences in *P. falciparum* infection between the primary malaria vectors *An. gambiae* s.s., *An. arabiensis* and *An. funestus* s.s. or between human fed and non-human fed mosquitoes.

**Table 4:** *Plasmodium falciparum* infection among engorged field collected anophelines

Study area	Mosquito species	n	HF	NHF	MF	UF	nPfal (%)	ERF (95% CI)
Mageta	*An. gambiae* s.s.	310	23	4	1	2	30 (9.68)	0.10 (0.067, 0.13)
	*An. arabiensis*	40	3	0	0	1	4 (10.00)	0.11 (0.032, 0.21)
	*An. funestus* s.s.	16	0	0	0	0	0 (0.00)	0.04 (3.9e−06, 0.16)
	*An. coustani*	8	1	0	0	1	2 (25.00)	0.29 (0.055, 0.57)
	Total	374	27	4	1	4	36 (9.63)	
Magare	*An. gambiae* s.s.	7	1	0	0	1	2 (28.57)	0.32 (0.059, 0.62)
	*An. arabiensis*	2	0	0	0	1	1 (50.00)	0.50 (0.085, 0.89)
	*An. funestus* s.s.	8	0	0	0	0	0 (0.00)	0.07 (5.4e−07, 0.28)
	*An. coustani*	8	0	1	0	0	1 (12.50)	0.18 (0.011, 0.44)
	Total	25	1	1	0	2	4 (16.00)	
Ngodhe	*An. gambiae* s.s.	3	0	0	0	0	0 (0.00)	0.16 (5.4e−07, 0.53)
	*An. arabiensis*	9	1	0	0	0	1 (11.11)	0.16 (0.0068, 0.39)
	*An. funestus* s.s.	5	0	0	0	0	0 (0.00)	0.11 (3.4e−06, 0.40)
	Total	17	1	0	0	0	1 (5.88)	
Total (%)		416	29 (6.97)	6 (1.44)	1 (0.24)	5 (1.20)	41 (9.86)	

n: number of engorged anophelines analysed; nPfal: overall number of engorged anophelines with *P. falciparum* infection confirmed by 18S and *cyt b* markers; HF: human-fed engorged anophelines with *P. falciparum* infection; NHF: non-human fed engorged anophelines with *P. falciparum* infection; MF: mixed-fed (human and cow) engorged anophelines with *P. falciparum* infection; UF: unsuccessfully identified blood-meal sources of engorged anophelines with *P. falciparum* infection; ERF: estimated relative frequency of success; CI: Credibility interval

## Discussion

Consistent with previous studies in other parts of the region [10, 11], this study identified four anopheline species, namely *An. gambiae* s.s., *An. arabiensis, An. funestus* s.s., and *An. coustani*. Overall, anophelines were most abundant in Mageta compared to Magare and Ngodhe, likely due to Mageta’s relatively low LLIN coverage. In all islands, the majority of engorged *An. gambiae* s.s., *An. arabiensis* and *An.* funestus s.s. were collected indoors by CDC light, revealing their tendency of feeding indoors, while *An. coustani* mosquitoes were collected predominantly outdoors. Further, the blood-meals from the species collected indoors were fresh, showing that they rested indoors after feeding [41], predominantly on cattle. Unlike a previous study in East Africa, which showed that *An. funestus* largely completes its developmental cycle indoors [42], there were fewer gravid anophelines, including *An. funestus*, collected with the resting traps (ASP and PSC) compared to the blood-fed vectors, indicating that gonotrophic development was completed outdoors.

Mosquitoes feed on potentially diverse hosts [2, 3, 33, 43]. Therefore, it is necessary to establish mosquito blood-feeding patterns to understand malaria transmission dynamics and provide strategies for optimal vector control. Vector control strategies depend largely on LLINs. However, their use is threatened by changes in mosquito behaviour and human behaviour/activities such as night fishing that alter disease transmission dynamics. Therefore, adequate knowledge of vector species and feeding patterns can inform the efficacy of and allow appropriate deployment of other vector control strategies, including LLINs.

Ten blood-meal hosts were identified by matching HRM profiles obtained using *COI*, *16S* rRNA and *cyt b* genes from engorged abdomens of field-collected mosquitoes to those obtained from standard reference positive controls and verified by sequencing. Although HRM analysis of the three distinct genetic markers could resolve a broad diversity of vertebrate hosts, this study did not detect host DNA from 7.21% of engorged abdomens and there is uncertainty why there were these amplification failures, despite the analysis involving the use of a shorter *COI* fragment (130-bp) that is suitable for degraded DNA samples from digested blood-meals [44]. The amplification failures could be potentially attributed to much older, degraded blood-meals. Nonetheless, this study demonstrates that anopheline mosquitoes in these small islands have a wide host range, able to sustain mosquito populations, and decrease transmission intensity. However, in the largest island, Mageta, LLIN coverage was low, allowing for increased human feeding indoors by *An.* gambiae s.s.*, An. arabiensis,* and *An. funestus* s.s, and contributing to transmission.

Overall, the data also show that *An. coustani* mosquitoes fed predominantly on cattle outdoors. In *An. gambiae* s.s, humans were the most common source of blood-meals; however, blood-meal sources also included diverse non-human hosts. Blood-meals from both *An. arabiensis* and *An. funestus* collected indoors were more substantially from humans, unlike in a study in Mwea which found significantly higher bovine feeding among indoor-collected *An. arabiensis*. Indoor-collected *An. funestus* also preferentially fed on humans [45]. Although a human blood-meal was identified in one *An. coustani* mosquito, which was also *P. falciparum* positive, this species preferred feeding outdoors on cows as previously shown [46].

Anopheline feeding patterns depend on the density and diversity of host species [47], which by their availability form readily accessible blood-meal sources. While PSC and ASP traps enabled us to understand anophelines’ endophily, by placing CDC traps indoors and outdoors that target host-seeking anophelines, this study aimed to maximize the recovery of data on feeding patterns (exophagy versus endophagy) to enhance understanding of localized anopheline feeding dynamics. Although the majority of trapping was done indoors, this study found that the range of host species can vary depending on study areas. Mageta had the highest numbers of engorged anophelines and broadest range of host species. In contrast, Ngodhe had the fewest engorged anopheline and narrowest range of host species as determined by PCR-HRM. The range of host species did not differ significantly in Magare compared to Ngodhe, perhaps reflecting lower vertebrate species diversity on these small islands compared to Mageta.

The blood-meal identification approach in this study allowed for high sensitivity identification of mixed blood-meals from individual mosquitoes. Mixed blood-meal sources could have been a result of blood-feeding anophelines resuming blood-feeding on a different host in an effort to complete an unsuccessful blood-meal, a characteristic that is common with anophelines infected with sporozoite-stage malaria parasites [48, 49]. The finding that mixed feeding on vertebrate hosts included blood-meals from cow, chicken and goat further confirmed malaria vectors feeding on readily available blood-meal sources [47], underlining the economic activities of the study population, which provide other blood-meal sources important for vector survival. However, the analysis was not able to differentiate multiple hosts of the same species within individual mosquito, blood-meals such as in The Gambia where one malaria vector was suspected to have taken blood-meals from children sharing rooms on one night [50]; this would require more extensive genotyping of blood-meals.

Overall, 9.86% of engorged anophelines harboured malaria parasites. There was no significant difference among engorged anopheline species in terms of *P. falciparum* infection rates among all four species or among the three islands. Further, neither island sizes nor blood-meal sources influenced *P. falciparum* infection rates in the vector. However, the vector species play an important role in malaria transmission by harbouring malaria parasites. These findings suggest that human-mosquito contact is still very frequent in the islands and has not been well controlled, therefore presenting a risk of malaria transmission.

## Conclusions

This study shows that on the small remote islands of Mageta, Magare and Ngodhe in Kenya’s Lake Victoria, anopheline mosquitoes are maintained by humans and, in part, by other blood-meal hosts. Among these islands, only Mageta had below WHO-recommended LLIN coverage, and significantly higher *Anopheles* mosquito abundances. The availability of alternative blood-meal sources not affected by LLINs can lead to opportunistic feeding events that present a challenge to malaria control efforts. This study indicates that there is an urgent need to target indoor-feeding mosquito populations by achieving full LLIN coverage. In addition, this is the first report of *An. coustani* with sporozoite infection in the area, highlighting the need to target secondary vectors with a more exophilic behaviour.

## Abbreviations

CI: credibility interval
ERF: estimated relative frequency of success
HRM: high-resolution melting
KEMRI: Kenya Medical Research Institute
LLINs: long-lasting insecticides nets
nPCR-HRM: nested polymerase chain reaction-high-resolution melting
PCR: polymerase chain reaction
PSC: pyrethrum spray catches
WHO: World Health Organization
