# Erratum to: Composition of *Anopheles* mosquitoes, their blood-meal hosts, and *Plasmodium falciparum* infection rates in three islands with disparate bed net coverage in Lake Victoria, Kenya

**DOI:** 10.1186/s12936-017-2030-6

**Published:** 2017-09-19

**Authors:** Edwin Ogola, Jandouwe Villinger, Danspaid Mabuka, David Omondi, Benedict Orindi, James Mutunga, Vincent Owino, Daniel K. Masiga

**Affiliations:** 1 International Centre of Insect Physiology and Ecology (icipe), P.O. Box 30772, Nairobi, 00100 Kenya; 2 Department of Biochemistry and Molecular Biology, Egerton University Njoro Campus, P.O. Box 536, Egerton, 20115 Kenya

## Erratum to: Malar J (2017) 16:360 DOI 10.1186/s12936-017-2015-5

After publication of the article [1], it has been brought to our attention that a funding acknowledgement has been omitted from the original article. The authors would like to include the following, “The study was undertaken as part of the Target Malaria consortium, which receives core funding from the Bill & Melinda Gates Foundation & from the Open Philanthropy Project Fund, an advised fund of Silicon Valley Community Foundation.”
